# Success and failure in narrowing the disability employment gap: comparing levels and trends across Europe 2002–2014

**DOI:** 10.1186/s12889-017-4938-8

**Published:** 2017-12-02

**Authors:** Ben Baumberg Geiger, Kjetil A. van der Wel, Anne Grete Tøge

**Affiliations:** 1 School of Social Policy, Sociology and Social Research (SSPSSR), University of Kent, Canterbury, Kent CT2 7NZ UK; 2 Department of Social Work, Child Welfare and Social Policy, Oslo and Akershus University College of Applied Sciences, Oslo, Norway; 3 Work Research Institute (AFI), Oslo and Akershus University College of Applied Sciences, Oslo, Norway

**Keywords:** Europe [Z01.542], Employment [N01.824.245], Policy [I01.655]; disability

## Abstract

**Background:**

International comparisons of the disability employment gap are an important driver of policy change. However, previous comparisons have used the European Union Statistics on Income and Living Conditions (EU-SILC), despite known comparability issues. We present new results from the higher-quality European Social Survey (ESS), compare these to EU-SILC and the EU Labour Force Survey (EU-LFS), and also examine trends in the disability employment gap in Europe over the financial crisis for the first time.

**Methods:**

For cross-sectional comparisons of 25 countries, we use micro-data for ESS and EU-SILC for 2012 and compare these to published EU-LFS 2011 estimates. For trend analyses, we use seven biannual waves of ESS (2002–2014) with a total sample size of 182,195, and annual waves of EU-SILC (2004–2014) with a total sample size of 2,412,791.

**Results:**

(i) *Cross-sectional*: countries that have smaller disability employment gaps in one survey tend to have smaller gaps in the other surveys. Nevertheless, there are some countries that perform badly on the lower-quality surveys but better in the higher-quality ESS. (ii) *Trends:* the disability employment gap appears to have declined in ESS by 4.9%, while no trend is observed in EU-SILC – but this has come alongside a rise in disability in ESS.

**Conclusions:**

There is a need for investment in disability measures that are more comparable over time/space. Nevertheless, it is clear to policymakers there are some countries that do consistently well across surveys and measures (Switzerland), and others that do badly (Hungary).

**Electronic supplementary material:**

The online version of this article (10.1186/s12889-017-4938-8) contains supplementary material, which is available to authorized users.

## Background

The disability employment gap – the difference in employment rates between disabled and non-disabled people – is a crucial indicator of disability equality. It is one measure of the right ‘to the opportunity to gain a living by work’, which forms Article 27 of the UN Convention on the Rights of Persons with Disabilities (ratified by the EU and the majority of Member States)*.* International comparisons of the gap also drive policy debate. Poor performance in OECD estimates [1] has been cited in Australia as a reason for reform [2], as has poor performance in the UK [3]. At a supranational level, failures to improve the disability employment rate were one of the criticisms of the EU’s European Action Plan 2004–10 [4].

However, these comparisons are beset by problems. Most of the few previous analyses of the disability employment gap – including by the OECD [1], the Commission’s Academic Network of European Disability Experts [5] and indeed ourselves [6] – have used the European Union Statistics on Income and Living Conditions, ‘EU-SILC’. Yet EU-SILC may be a less reliable basis for strong conclusions [7], because the EU Regulation underpinning it requires a common set of *outputs* to be produced, but does not require the *process* of collecting these to be consistent. Countries therefore differ in question wording, survey mode, and their use of proxy interviews (for example, 13 countries in 2011 used only face-to-face interviews, but 4 countries used predominantly telephone interviews [8]; while proxy rates in 2009 vary from 1% in Sweden to 48% in Denmark [9]), all of which influence health reporting [10]. One paper [11] has used another EU survey, the European Labour Force Survey (‘EU-LFS’), where identical issues apply.

A high-quality survey with comparable methods across countries does exist – the European Social Survey (‘ESS’) – but has not previously been used. Our first contribution is therefore to present the disability employment gap in ESS for the first time. We also compare the ESS estimates to those from EU-SILC and the EU LFS, given evidence that different surveys can show different cross-national comparisons [10] and within-country trends [12, 13] in health/disability.

Our final contribution is to examine trends in the disability employment gap using ESS and EU-SILC. Almost no trend analyses have previously been published, with a particularly absence of studies comparing any changes since the 2007 financial crisis in Europe. (Crisis-focussed studies using EU-SILC exist, but these focus on unemployment among disabled people [14, 15], and may therefore capture movements between unemployment and inactivity). The most influential previous trend study of disability employment is by the OECD [1], but this not only uses the EU-SILC for the most recent period, but compares this to the earlier European Community Household Panel, despite the methods and measures of the two studies being different by design [16]. No other studies of trends in the disability employment gap exist, other than a brief analysis of short-run EU-SILC trends 2008–2012 [5], and a comparative study using non-comparable but high-quality national surveys [17].

### Measuring disability

There are many defensible ways of measuring disability, and these produce different reported levels of disability [18, 19]. This influences the disability employment gap – broader definitions of disability will tend to capture more people with milder disabilities, and therefore produce smaller gaps [13]. The conventional way that disability employment gaps are measured is by asking people if they have a longstanding health condition or disability that limits their day-to-day activities, and this is the question that we use below.

However, the difficulty in making comparisons across time/countries is that the reporting of disability will not be consistent, and that apparent differences in gaps may simply reflect changes in disability reporting. This is inherent to defining disability as a restriction on participating in social roles [20]: environmental factors will be more/less disabling in different times and places. At extremes, successful policies may produce *widening* gaps, to the extent they cause people with milder impairments to no longer say that this affects their day-to-day lives – a risk accepted by the UK Government in their monitoring of the upcoming disability employment strategy [21].

The task of creating more comparable disability measures is being led by the Washington Group on Disability Statistics (a UN agency), who propose using functional limitation measures as indicators of *potential* disability [22]. However, these are not currently available within robust comparative datasets. To respond to this issue, we therefore consider disability *prevalence* when interpreting disability employment figures, and also propose combining prevalence & employment gaps into a single measure, as we explain below.

## Methods

### ESS data

This paper primarily focuses on waves 1–7 of ESS [23–29], which makes the strongest efforts to achieve comparability of any repeated cross-national survey [30]. Question wording is extensively checked, proxies are not allowed, and survey modes & sampling methodologies are consistent. We exclude non-EU/EEA member countries and countries that do not collect data for at least two time points, leaving 25 countries. While we focus on the 15–64 population for comparison to published EU-LFS tables, in the trend analyses we focus on the 25–64 working-age population (to avoid differences in higher education policy), with sample sizes per country-wave of 579–3045 across 150 country-wave observations, and a total sample of 182,195. Response rates in each ESS round vary from 30 to 80%; we use post-stratification weights to adjust for sampling error and non-response bias, and population weights when summarising across multiple countries.

Disability in ESS is measured by asking, *“Are you hampered in your daily activities in any way by any longstanding illness, or disability, infirmity or mental health problem?”* (We combine those saying they are hampered ‘a lot’ and ‘to some extent’). Employment status is measured by asking, *“Using this card, which of these descriptions applies to what you have been doing for the last 7 days? Select all that apply.”* We define someone as working if they give the answer *‘in paid work (or away temporarily) (employee, self-employed, working for your family business)’*.

### Other data

For the purposes of comparison, we also use EU-SILC and the 2011 ad-hoc module of the EU-LFS. Both surveys are multi-wave rotating panel designs that are governed by EU Regulations (EU-SILC by 1983/2003, EU-LFS by 317/2010) that specify minimum requirements, but allow some latitude in wording and methodology. For example, while most EU-SILC countries use face-to-face interviews, Iceland, the Netherlands, Finland, Denmark, Slovenia, Austria, Lithuania, Latvia, Spain and Greece to a varying extent use telephone interviews [8], which will influence disability reporting [10]. Similarly, proxy interviews in EU-SILC vary from <10% in Sweden to nearly 50% in Denmark. Exactly the same issues arise in the EU-LFS (e.g. proxy interviews range from >50% in Slovenia to zero in Belgium) [31]. For both EU-SILC and EU LFS, no consistent information is available on response rates.

A key advantage of the EU-SILC is its large sample size: 1913 to 34,374 per country-wave, which for 2004–2014 results in a dataset of 2,412,791 observations nested in 258 country-years [32]. (The EU-LFS is similarly large, with country-waves varying between 3200 and 130,800). Unlike for the ESS and EU-SILC, we use the published aggregate EU-LFS data from Eurostat rather than microdata. Details of the definitions of disability and employment for both are given in Web Additional file 1: Appendix A1.

### Analytical plan

The analysis is divided into two stages. Firstly, we look at country differences in the disability employment gap in ESS, EU-SILC and the EU-LFS in 2011/12 (the year for which all three are available), and test for country differences in ESS & EU-SILC over the full 2002–2014 period. We also compare disability per se across counties, to see if countries that seem to have broader definitions of disability tend to have narrower employment gaps. As a further measure, we look at the product of disability prevalence and the disability employment gap within each country, which Berthoud [33] (and also Jones & Wass [34]) have proposed as a measure of ‘the number of people prevented from working by disability’. This does not completely overcome different interpretations of disability questions across time and space, but it does offer some way of capturing the combined effect of differences in disability prevalence and employment.

Secondly, we investigate trends in the disability employment gap in ESS and EU-SILC. We perform a regression analysis that adjusts for compositional changes in the European population (including age, gender, education, migrant status, living with partner, and any children in the household; further details are given in Additional file 1: Appendix A1). Because logit models are often misinterpreted [35], and linear regression for *common* binary outcomes is equally robust [36], we use linear (OLS) regression models; logit sensitivity analyses are supplied in Additional file 1: Appendix A3 and show similar results. Similarly, because the partitioning of variance between the individual and societal level is not of interest here, we use cluster-robust regression models; multilevel models in Additional file 1: Appendix A3 again give similar results.

## Results

### Which countries have the smallest disability employment gaps?

#### The gap in 2011–12

Countries’ rankings in the disability employment gap across the different surveys in 2011/12 is shown in columns 2–4 of Table 1 below. (Rankings are used for ease of comparability across surveys, but the estimates themselves are given in Additional file 1: Appendix Table A1).

**Table 1:** Countries’ rankings in disability employment across different surveys, 2011/12

	Ranking for gap			Ranking for rate		
	*ESS*	*EU-SILC*	*EU-LFS*	*ESS*	*EU-SILC*	*EU-LFS*
Austria		11	6		7	**5**
Belgium	20	22	15	19	20	15
Bulgaria	21	16	19	22	18	20
Cyprus	11	**4**	14	15	8	12
Czech Republic	16	23	16	10	19	16
Denmark	**2**	15	21	**5**	12	11
Estonia	**4**	14	10	**4**	10	9
Finland	8	6	**3**	6	**4**	**4**
France	9	**3**	**2**	9	**5**	6
Germany	**5**	9	11	7	**3**	7
Greece		8	13		23	17
Hungary	23	24	22	23	24	22
Iceland	15	20	9	14	16	**2**
Ireland	6	19	19	20	25	21
Italy	**1**	**1**	**5**	**1**	6	13
Netherlands	22	17		12	9	
Norway	17	25		8	13	
Poland	14	18	17	13	21	18
Portugal	18	13	8	21	17	8
Slovakia	19	**5**	18	17	11	19
Slovenia	12	10	12	18	15	10
Spain	10	12	7	16	22	14
Sweden	**3**	7	**1**	**3**	**2**	**3**
Switzerland	7	**2**	**4**	**2**	**1**	**1**
UK	13	21		11	14	

Bold numbers signifies the five countries in each survey with the smallest disability employment gap or the highest disability employment rate. Rank 1 is for best performance. Data is for 2011 in EU LFS and 2012 for ESS and EU-SILC

Looking across all the surveys, Switzerland, Sweden and Finland are consistently highly-ranked, while Belgium, the Czech Republic, Norway and especially Hungary perform consistently poorly. It is worth noting that the lowest gap in ESS is in Italy, where disabled people have 5.4% *higher* employment than non-disabled people. This is an outlier, but neither ourselves nor the Italy ESS team have been able to establish a specific error, and the same pattern is seen in 2004; moreover, while different in magnitude, we can see that Italy also has the lowest disability gap in EU-SILC, and one of the lowest in EU-LFS.

There are also some differences between the surveys, such as for Ireland and particularly Denmark (which is one of the best-performing countries in ESS, mid-to-low ranked in EU-SILC, and one of the worst-performing in EU-LFS). Overall, the agreement between pairs of surveys – measured by the absolute intra-class correlation coefficient – is high (0.72, *p* < 0.001). If we plot mean estimates against inter-survey differences for each pair of surveys in each country (known as Bland-Altman plots [10] and shown in Additional file 1: Appendix A2), then we can further see that there is no systematic pattern to the inter-survey differences with respect to the size of the disability employment gap .

**Fig. 1:**
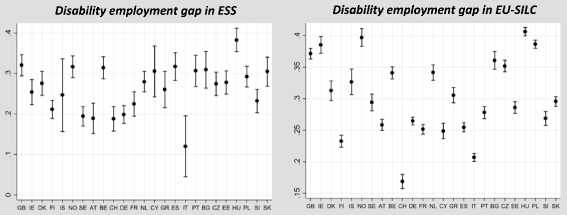
Disability employment gaps across Europe in ESS (2002–2014, left panel) and EU-SILC (2004–2014, right panel)

Some of these differences will be driven by sampling error, particularly for rankings [37]. To examine systematic differences in disability employment gaps between countries, we pool all the available survey years 2002–2014 and estimate confidence intervals around the disability employment gap in ESS & EU-SILC, shown in Fig. 1. This highlights that there are considerable differences between surveys, and moreover, the story they tell is different. In EU-SILC, there is no overall pattern for certain parts of Europe to have higher/lower gaps. In ESS, however, the lowest rates (excluding the outlier of Italy) are found in most of the central European and Nordic countries. Policymakers using EU-SILC may therefore take different lessons to those using the higher-quality ESS data.

#### Disability equality or methodological artefact?

One way that these results may be misleading is that they focus on *gaps* in employment rates, but it is not necessarily true that the countries with the lowest gaps have the highest levels of disability employment. Looking at columns 4–6 of Table 1 (and Additional file 1: Appendix Table A1), we can see that Norway has a noticeably higher disability employment *gap* than Spain (21.5% vs. 14.8% in ESS), but this reflects the much higher overall *level* of employment in Norway, and the employment rate for disabled people is actually higher in Norway (51.6% vs. 41.0%). Such distinctions influence the position of several countries, but the overall agreement between the two measures is still strong (when coded in the same direction, intra-class correlations are 0.72–0.86).

A further possibility is that the countries with the lowest disability employment gaps are simply those where more people say they are disabled (thereby including more people with less severe disabilities, as discussed above). Additional file 1: Appendix Figure A4 does indeed show that there is a negative relationship between disability prevalence and the disability employment gap, such that for each additional 1% of the population that reports a disability, the employment gap declines by 0.38%. Overall this is only a weak relationship, with a wide scattering of the observations around the trendline (the R^2^ is only 0.07). However, this is primarily because there is no relationship between prevalence and employment gaps in the highest-quality survey, ESS; within EU-SILC and particularly EU-LFS, there is a negative relationship (*r* = -0.05, -0.26 and -0.53 respectively).

If we look at the number of people prevented from working due to disability (shown in Additional file 1: Appendix Table A2) – Berthoud’s suggested measure estimated as the product of disability prevalence and the employment gap – then we see slightly different rankings once more. In ESS, Spain becomes the third best-performing country (compared to 10th when looking at the disability employment gap), with Germany looking worse (falling from fifth to 13th). Overall, this measure shows similar rankings to the disability employment gap for the highest-quality survey, ESS (*r* = 0.81), but greater differences for the other surveys (*r* = 0.34 and 0.52 for EU-SILC and EU-LFS respectively).

### Has the disability employment gap been narrowing or widening?

The regression model showing trends in employment and disability is shown in Table 2. The coefficient on ‘trend’ represents the employment trend among disabled people, and the coefficient on the interaction term ‘no disability * trend’ represents trends in the employment gap (the difference in trends between disabled and non-disabled people).

**Table 2:** Trends in employment among disabled people 2002–2014

	ESS		EU-SILC	
	Model 1	Model 2	Model 3	Model 4
No disability	0.280***	0.210***	0.263***	0.183***
Trends among disabled people^a^				
Pre-2006	(Baseline)	(Baseline)	(Baseline)	(Baseline)
2006–2011	0.051***	0.040**	−0.008	−0.013
Post-2011	0.089***	0.076***	0.027	0.026
Trends in disability employment gap^a^				
No disability * 2006–2011	−0.013	−0.008	0.030*	0.030*
No disability * Post-2011	−0.057***	−0.049***	0.011	0.004
Adjustment for compositional factors^b^	No	Yes	No	Yes
*Observations*	*182,195*	*182,195*	*2,412,791*	*2,412,791*

^a^Years differ in ESS and EU-SILC due to data availability: periods are split into early (2002–4 ESS, 2004 EU-SILC), recent (2012–2014), and an intermediate period (2005–2011 EU-SILC, 2006–2010 ESS)

^b^Adjustment uses the following covariates: age, gender, education, migrant status, living with partner, and any children in the household. Coefficients on these covariates are given in Additional file 1: Appendix Table A3

*** *p*  <0.01, ** *p*  <0.05, * *p*  <0.1

Looking first at ESS without adjustment for compositional changes (Model 1), we see that there has been a considerable increase in the employment rates of disabled people in Europe, by 0.089 (8.9%) pre-2006 to post-2011. These trends are similar when we adjust for compositional changes (a rise of 7.6% in Model 2). Moreover, if we look at the interaction terms below that capture the changing disability employment gap, we see that this also represents a reduction in the disability employment gap of 5.7% from pre-2006 to post-2011 (4.9% after accounting for compositional changes). We might expect disabled people to be protected from layoffs in times of recession due to disability discrimination protection (or employment protection more generally) [15], but it is striking that the shrinking gap in ESS comes from a sharp rise in disability employment.

Turning to the equivalent models for EU-SILC, we again see an increase in employment among disabled people (Model 3). However, this is smaller than ESS and non-significant (2.7% without adjustment, 2.6% with adjustment). Moreover, looking at the trends in the disability employment *gap*, there is no sign of any improvement in the relative labour market position of disabled people across the financial crisis (indeed, there are non-significant increases of 1.1% without adjustment and 0.4% with adjustment). In other words, we see a marked improvement in disability equality in the labour market in one dataset (ESS), but no change in the other (EU-SILC).

To investigate possible explanations for this, we look at trends in disability per se in the two surveys, which is shown in Table 3. This shows a statistically significant rise in reported disability in ESS of noticeable size (2.0% higher in post-2011, compared to a prevalence of 18.4% at baseline (not reported)). Yet there is little sign of any change in disability in EU-SILC. This difference between surveys is not reconciled if we use the alternative measure of the percentage prevented from working due to disability (across the EU, ESS shows a fall from 5.2 to 4.5%, while there is a smaller *increase* in EU-SILC from 4.8 to 5.0%).

**Table 3:** Trends in disability in Europe 2002–2014

	ESS		EU-SILC	
	Model 1	Model 2	Model 3	Model 4
Trends^a^				
Pre-2006	(Baseline)	(Baseline)	(Baseline)	(Baseline)
2006–2011	0.005	0.006	−0.008	−0.008
Post-2011	0.020***	0.021***	0.003	0.003
Adjustment^b^	No	Yes	No	Yes
*Observations*	*182,195*	*182,195*	*2,412,791*	*2,412,791*

^a^Years differ in ESS and EU-SILC due to data availability: periods are split into early (2002–4 ESS, 2004 EU-SILC), recent (2012–2014), and an intermediate period (2005–2011 EU-SILC, 2006–2010 ESS)

^b^Adjustment uses the following covariates: are age, gender, education, migrant status, living with partner, and any children in the household. Coefficients on these covariates are given in Additional file 1: Appendix Table A4

*** *p*  <0.01, ** *p*  <0.05, * *p*  <0.1

We also perform a variety of sensitivity analyses, including different definitions of employment, using a continuous rather than categorical trend term, using multilevel models rather than cluster-robust standard errors, and using logit rather than OLS. While some results change slightly, our main results – the narrowing disability employment gap in ESS, but smaller & less consistent trends in EU-SILC – are robust; full details are given in Additional file 1: Appendix A3.

## Discussion

While the disability employment gap is an important indicator of success in including disabled people in the labour market, the only previous international comparisons have used EU-SILC or EU-LFS data. These have questionable validity, given known and sizeable differences in question wording, response modes, and use of proxies. In this survey, we present results from a survey with consistent wording, response mode and use of proxies across countries (ESS) for the first time, and compare this to EU-SILC and EU-LFS.

We find that countries that have smaller disability employment gaps in one survey tend to have smaller gaps in the other surveys – but the surveys also differ in ways that cannot be explained by sampling error. Some countries (such as Switzerland and Finland) do consistently well, while others (especially Hungary) do consistently poorly. However, there are countries like Denmark and Ireland that perform well (i.e. have relatively small disability employment gaps) in ESS, but perform less well in the lower-quality surveys. Moreover, in ESS it tends to be Central European and Nordic countries that have the smallest gaps, while there are no such systematic patterns visible in EU-SILC.

We also use ESS to investigate trends in the disability employment gap across the recent economic crisis, which has not previously been studied. We find a marked improvement in disability equality in the labour market in ESS, with disability employment rising by 7.6% pre-2006 to post-2011 (net of compositional changes), and the disability employment gap falling by 4.9% - although a 22% disability employment gap remains. In contrast, there is no overall change in the disability employment gap across Europe in EU-SILC in the main analyses and most of the sensitivity analyses (while one sensitivity analysis shows a declining gap, the effect is nevertheless much smaller).

However, even with noticeably higher-quality data, there remains an issue about whether ‘disability’ is interpreted consistently across time and space. If we look at the trends in disability per se, we see an unexplained rise in disability reporting in ESS (by 2.1% pre-2006 to post-2011, net of compositional changes), which again is not found in EU-SILC. It is possible to create an overall measure of the proportion of people prevented from working by disability, using the product of the disability prevalence rate and the disability employment gap (as suggested by Berthoud and Wass & Jones). On this measure, we still see an improvement over time in ESS, yet not in EU-SILC.

## Conclusion

In conclusion, there is a need for investment in disability measures that are more comparable over time and space. Nevertheless, there is some agreement between surveys and measures that some countries (e.g. Switzerland) do consistently well, and others (e.g. Hungary) doing consistently badly. The high-quality ESS data presented here for the first time also produce a slightly different picture from previous surveys, with Denmark and Ireland doing better than had previously been suggested, and many countries in Central Europe and the Nordic countries (as well as Italy) having the smallest disability employment gaps.

Future research should investigate the role of social policies and labour market conditions in shaping these patterns of disability employment – as indeed we ourselves will do in a future companion paper. In the meantime, policymakers may want to look at the policy mix in countries that appear to be consistently successful (e.g. Switzerland) for ideas of how to reduce the inequality between disabled and non-disabled people in the labour market.

## Additional file

Additional file 1: Online-only appendices. (DOC 357 kb)

## Abbreviations

ESS: European social survey
EU: European Union
EU-LFS: EU Labour Force Survey
EU-SILC: European union statistics on income and living conditions
OECD: Organisation for economic co-operation and development
OLS: Ordinary least squares regression
UN: United Nations
