# Supplementary material for: Success and failure in narrowing the disability employment gap: comparing levels and trends across Europe 2002–2014
Source: BMC Public Health. 2017 Dec 2;17:928. doi: 10.1186/s12889-017-4938-8 (PMC5712075; doi:10.1186/s12889-017-4938-8)
Supplement: Additional file 1: — Online-only appendices. (DOC 357 kb) [file 12889_2017_4938_MOESM1_ESM.doc]

# Supplementary material:

# Appendices for *‘Success and failure in narrowing the disability employment gap: comparing levels and trends across Europe 2002-2014’*

## Appendix A1: Variable details not given in the main paper

### Disability and employment comparisons (Table 1)

#### Disability (EU-SILC & EU-LFS)

Because of the variation in data collection in EU-SILC, the measures of disability and employment are harmonized to the extent that countries are required to measure certain concepts, even if the wording is not consistent. For disability, countries are required to measure *‘limitation in activities people usually do because of health problems for at least the last six months’* – a similar (but not identical) concept to that used in ESS*.* As with ESS, we define both people replying ‘yes, strongly limited’ and ‘yes, limited’ as disabled.

The measure of disability from the LFS is slightly different to ESS/EU-SILC. Respondents were asked if they had any of a list of basic activity limitations (e.g. ‘sitting or standing’); following Eurostat, we define someone as disabled if they report any of these limitations. Some countries (the Netherlands, Norway and the UK) only asked this to people after an initial filter question, counter to Eurostat’s recommendation; this is likely to reduce the reporting of disability in these countries and they are therefore excluded from analysis below.

#### Employment (EU-SILC & EU-LFS)

In EU-SILC, countries are required to measure respondent’s ‘self-defined current economic status’. We consider respondents to be ‘employed’ if they report being full- or part-time employees, self-employed or family workers.

In EU-LFS, the measure of employment is the standard Eurostat employment measure of people who have worked for 1+hrs in the past week (including those who were temporarily absent).

### Control variables for ESS & EU-SILC trend regressions (Tables 2 & 3)

#### Education

In ESS, education was measured by the highest level of education. ESS includes a variable (edulvla), based on ISCED-97 (UNESCO 1997[[1]](#footnote-2)), that has been harmonised across all waves (ESS 2017[[2]](#footnote-3)). Education was coded into three levels; short (lower secondary or less), intermediate (upper-secondary and post-secondary, non-tertiary education) and tertiary education.

In EU-SILC, education was measured by the highest level of education. EU-SILC includes a variable PE040 (Highest ISCED level attained ), which was coded into three levels; short (lower secondary or less), intermediate (upper-secondary and post-secondary, non-tertiary education) and tertiary education.

#### Other variables: age, gender, migrant status, living with partner, children in household

In ESS, gender was coded 1 if female, and otherwise 0. Age was centred on its mean and used as a continuous measure. Immigrant status was measured by a dummy indicator taking the value 1 for people not born in the country of the survey. Through ESS’s household grid, we also identified respondents living with their partner and the presence of children in the household, both coded 1 on dummy variables.

In EU-SILC, gender was coded 1 if female, and otherwise 0. Age was centred on its mean and used as a continuous measure. Immigrant status was measured by a dummy indicator taking the value 1 for people not born in the country of the survey. Through EU-SILC’s household files, we also identified respondents living with their partner and the presence of children in the household, both coded 1 on dummy variables.

### Weights

For both ESS and EU-SILC, we apply a weight that adjusts for design (i.e. unequal chances of selection), non-response, and population size:

- In ESS, this is the combination of the design weight PWEIGHT and population size weight PSPWGHT.
- In EU-SILC, this is the combination of the design weight provided within the data file (RB050), while the population weight is the calculated by the authors (population size divided on number of respondents). A product of these two weights is used as a combined weight in the final analyses.

## Appendix A2: Additional results

#### Disability employment levels & gaps in each country

Table 1 in the main paper shows the *ranking* of the disability employment gap in each country – Table A1 below shows the actual values that underpin this.

Table A1: Countries’ disability employment rates and gaps across different surveys, 2011/12

***Disability employment gap***

***Disability employment rate***

|  | ESS | EU-SILC | EU-LFS |  |  | ESS | EU-SILC | EU-LFS |
| --- | --- | --- | --- | --- | --- | --- | --- | --- |
| Austria |  | 22.0% | 15.3% |  |  |  | 49.0% | 60.3% |
| Belgium | 24.1% | 31.3% | 25.7% |  |  | 38.4% | 36.3% | 40.7% |
| Bulgaria | 25.0% | 24.3% | 31.1% |  |  | 33.9% | 40.1% | 30.7% |
| Cyprus | 15.5% | 16.9% | 24.5% |  |  | 41.3% | 48.9% | 46.4% |
| Czech Republic | 20.6% | 32.0% | 29.9% |  |  | 46.9% | 38.1% | 38.6% |
| Denmark | 9.4% | 23.3% | 31.4% |  |  | 55.0% | 45.5% | 46.7% |
| Estonia | 9.7% | 22.9% | 19.1% |  |  | 58.1% | 48.1% | 49.5% |
| Finland | 11.9% | 17.7% | 12.4% |  |  | 54.1% | 52.8% | 60.8% |
| France | 14.8% | 16.1% | 9.9% |  |  | 47.6% | 52.0% | 56.2% |
| Germany | 10.9% | 21.3% | 20.6% |  |  | 53.1% | 53.3% | 51.5% |
| Greece |  | 21.1% | 23.0% |  |  |  | 32.7% | 35.5% |
| Hungary | 28.8% | 33.6% | 37.4% |  |  | 31.4% | 27.8% | 23.7% |
| Iceland | 20.5% | 30.5% | 17.1% |  |  | 42.2% | 44.1% | 66.9% |
| Ireland | 11.6% | 30.3% | 31.1% |  |  | 37.1% | 26.9% | 29.8% |
| Italy | -5.4% | 10.5% | 13.3% |  |  | 64.9% | 49.0% | 45.6% |
| Netherlands | 25.5% | 27.9% |  |  |  | 44.0% | 48.5% |  |
| Norway | 21.5% | 35.7% |  |  |  | 51.6% | 45.3% |  |
| Poland | 18.7% | 29.6% | 30.0% |  |  | 43.6% | 36.1% | 33.9% |
| Portugal | 21.5% | 22.8% | 16.8% |  |  | 35.1% | 41.4% | 51.0% |
| Slovakia | 21.8% | 17.7% | 30.7% |  |  | 39.7% | 46.7% | 31.9% |
| Slovenia | 16.1% | 21.7% | 21.4% |  |  | 38.6% | 44.4% | 47.0% |
| Spain | 14.8% | 22.2% | 16.2% |  |  | 41.0% | 34.7% | 44.3% |
| Sweden | 9.5% | 20.8% | 9.5% |  |  | 59.2% | 57.1% | 66.2% |
| Switzerland | 11.8% | 15.2% | 12.6% |  |  | 60.4% | 64.1% | 69.0% |
| UK | 18.4% | 30.8% |  |  |  | 46.3% | 44.5% |  |

Notes: Rank 1 is for best performance (i.e. the smallest gap and the highest employment rate). Data is for 2011 in EU LFS and 2012 for ESS and EU-SILC.

#### Bland-Altman plots

These plots show the mean estimates of the disability employment gap across each pair of surveys, plotted against the differences between that pair of surveys. These are used similarly by Croezen et al 2016 [1] to examine whether differences between surveys are more likely to occur at higher or lower values. As can be seen in the three plots below, there is no telling evidence of systematic differences between each pair of surveys.

Figure A1: Bland-Altman plot of ESS vs. EU-SILC


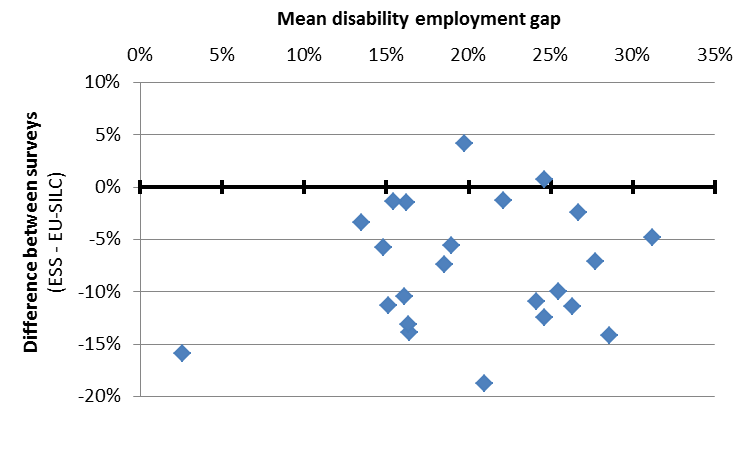


Figure A2: Bland-Altman plot of ESS vs. EU-LFS


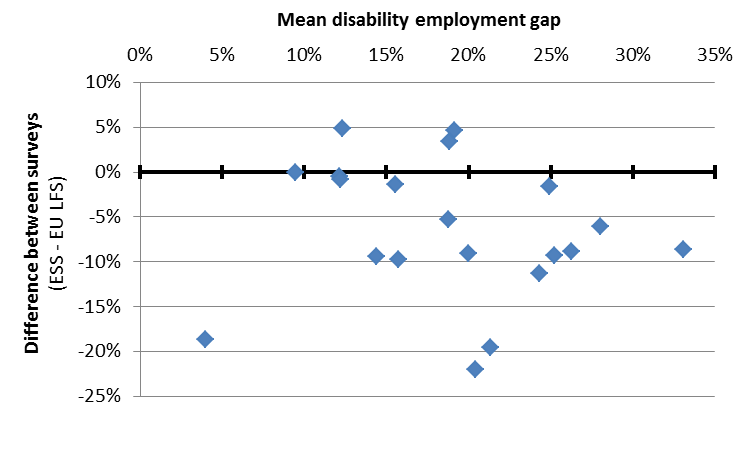


Figure A3: Bland-Altman plot of EU-SILC vs. EU-LFS


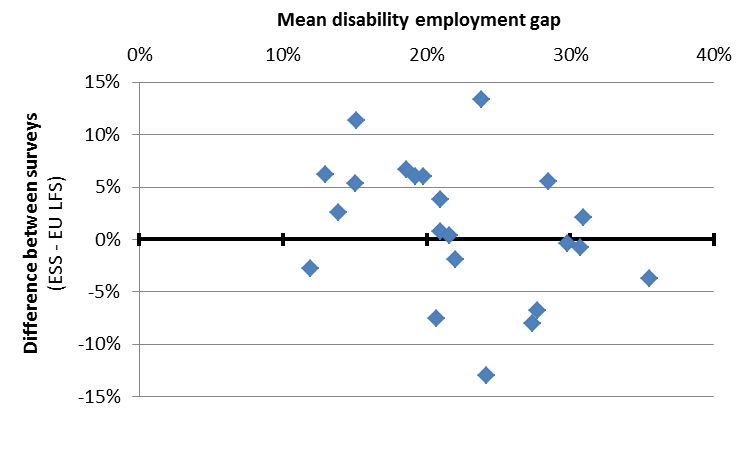


#### The country-level relationship of disability prevalence to the disability employment gap

In the main text, we note that *“there is a negative relationship between disability prevalence and the disability employment gap, such that for each additional 1% of the population that reports a disability, the employment gap declines by 0.38%. Overall this is only a weak relationship, with a wide scattering of the observations around the trendline (the R^2^ is only 0.07). However, this is primarily because there is no relationship between prevalence and employment gaps in the highest-quality survey, ESS; within EU-SILC and particularly EU-LFS, there is a negative relationship (r=-0.05, -0.26 and -0.53 respectively).”*

The plot underpinning these results is shown in Figure A4 below.

Figure A4: Relationship of disability prevalence to disability employment gap


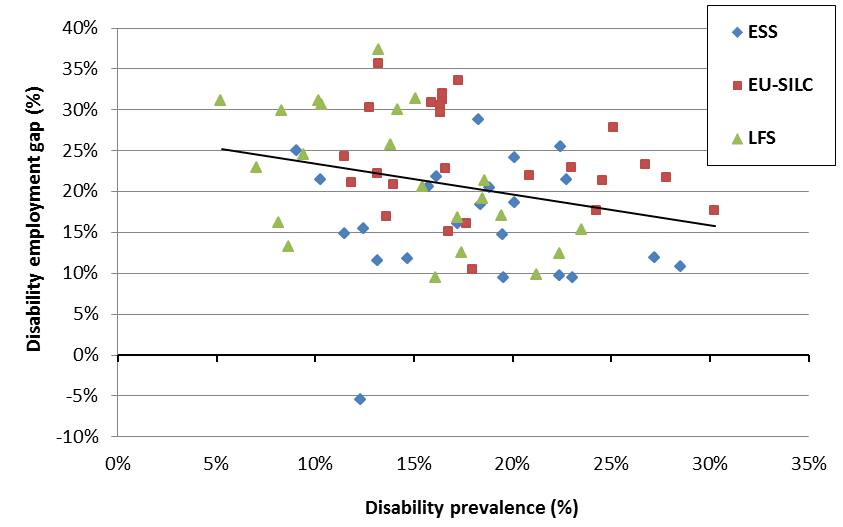


#### The number of people prevented from working due to disability

In the main text, we refer to a further measure that looks at the product of disability prevalence and the disability employment gap within each country, which Berthoud [2] (and also Jones & Wass [3]) have proposed as a measure of ‘the number of people prevented from working by disability’. This is discussed in the main text, and the full results are shown in Table A2 below.

Table A2: The number of people prevented from working due to disability^1^, 2011/12

***Number prevented from working***

***Ranking on this measure***

|  | ESS | EU-SILC | EU-LFS |  |  | ESS | EU-SILC | EU-LFS |
| --- | --- | --- | --- | --- | --- | --- | --- | --- |
| Austria |  | 4.6% | 3.6% |  |  |  | 12 | 18 |
| Belgium | 4.9% | 5.1% | 3.6% |  |  | 20 | 17 | 17 |
| Bulgaria | 2.3% | **2.8%** | 3.2% |  |  | 10 | **5** | 13 |
| Cyprus | 1.9% | **2.3%** | 2.3% |  |  | 6 | **2** | 8 |
| Czech Republic | 3.2% | 5.3% | 2.5% |  |  | 15 | 20 | 9 |
| Denmark | **1.9%** | 6.2% | 4.8% |  |  | **5** | 24 | 21 |
| Estonia | 2.2% | 5.3% | 3.5% |  |  | 7 | 19 | 16 |
| Finland | 3.2% | 5.4% | 2.8% |  |  | 14 | 21 | 10 |
| France | 2.9% | 2.8% | 2.1% |  |  | 12 | 6 | 6 |
| Germany | 3.1% | 5.2% | 3.2% |  |  | 13 | 18 | 14 |
| Greece |  | **2.5%** | **1.6%** |  |  |  | **3** | **4** |
| Hungary | 5.3% | 5.8% | 5.0% |  |  | 22 | 22 | 22 |
| Iceland | 3.9% | 5.0% | 3.3% |  |  | 19 | 16 | 15 |
| Ireland | **1.5%** | 3.9% | **1.6%** |  |  | **2** | 10 | **5** |
| Italy | **-0.7%** | **1.9%** | **1.2%** |  |  | **1** | **1** | **1** |
| Netherlands | 5.7% | 7.0% |  |  |  | 23 | 25 |  |
| Norway | 4.9% | 4.7% |  |  |  | 21 | 13 |  |
| Poland | 3.8% | 4.8% | 4.3% |  |  | 18 | 14 | 20 |
| Portugal | 2.2% | 3.8% | 2.9% |  |  | 9 | 9 | 11 |
| Slovakia | 3.5% | 4.3% | 3.2% |  |  | 17 | 11 | 12 |
| Slovenia | 2.8% | 6.0% | 4.0% |  |  | 11 | 23 | 19 |
| Spain | **1.7%** | 2.9% | **1.3%** |  |  | **3** | 8 | **2** |
| Sweden | 2.2% | 2.9% | **1.5%** |  |  | 8 | 7 | **3** |
| Switzerland | **1.7%** | **2.5%** | 2.2% |  |  | **4** | **4** | 7 |
| UK | 3.4% | 4.9% |  |  |  | 16 | 15 |  |

^1^ Number of people prevented from working due to disability is the product of the disability employment gap and disability prevalence in a particular country (see text).

Notes: Rank 1 is for best performance (i.e. the smallest gap and the highest employment rate). Data is for 2011 in EU LFS and 2012 for ESS and EU-SILC.

#### Coefficients on control variables in trend analyses

Tables 2 and 3 in the main paper show trends in disability employment and disability itself in ESS and EU-SILC. Models 2 and 4 adjust for compositional changes – the coefficients on the control variables (alongside the results as shown in the main paper) are shown in the tables below:

Table A3: Trends in employment among disabled people 2002-2014

|  | **ESS** | | **EU-SILC** | |
| --- | --- | --- | --- | --- |
|  | Model 1 | Model 2 | Model 3 | Model 4 |
| No disability | 0.280*** | 0.210*** | 0.263*** | 0.183*** |
| **Trends among disabled people^1^** |  |  |  |  |
| Pre-2006 | (Baseline) | (Baseline) | (Baseline) | (Baseline) |
| 2006-2011 | 0.051*** | 0.040** | -0.008 | -0.013 |
| Post-2011 | 0.089*** | 0.076*** | 0.027 | 0.026 |
| **Trends in disability employment gap^1^** |  |  |  |  |
| No disability * 2006-2011 | -0.013 | -0.008 | 0.030* | 0.030* |
| No disability * Post-2011 | -0.057*** | -0.049*** | 0.011 | 0.004 |
| **Controls** | No | Yes | No | Yes |
| Female |  | -0.166*** |  | -0.119*** |
| Age |  | -0.007*** |  | -0.008*** |
| Age^2^ |  | -0.001*** |  | -0.001*** |
| Any children in household |  | -0.037*** |  | -0.061*** |
| Not living with partner |  | -0.036*** |  | -0.058*** |
| Nor born in country |  | -0.066*** |  | -0.068*** |
| **Education** |  |  |  |  |
| High education |  | (Baseline) |  | (Baseline) |
| Intermediate education |  | -0.080*** |  | -0.083*** |
| Low education |  | -0.193*** |  | -0.207*** |
| *Observations* | *182,195* | *182,195* | *2,412,791* | *2,412,791* |

^1^ Years differ in ESS and EU-SILC due to data availability: periods are split into early (2002-4 ESS, 2004 EU-SILC), recent (2012-2014), and an intermediate period (2005-2011 EU-SILC, 2006-2010 ESS).

Table A4: Trends in disability in Europe 2002-2014

|  | **ESS** | | **EU-SILC** | |
| --- | --- | --- | --- | --- |
|  | Model 1 | Model 2 | Model 3 | Model 4 |
| **Trends^1^** |  |  |  |  |
| Pre-2006 | (Baseline) | (Baseline) | (Baseline) | (Baseline) |
| 2006-2011 | 0.005 | 0.006 | -0.008 | -0.008 |
| Post-2011 | 0.020*** | 0.021*** | 0.003 | 0.003 |
| **Controls** | No | Yes | No | Yes |
| Female |  | 0.018*** |  | 0.018*** |
| Age |  | 0.007*** |  | 0.007*** |
| Age^2^ |  | 0.000** |  | 0.000*** |
| Any children in household |  | -0.019*** |  | -0.019*** |
| Not living with partner |  | 0.051*** |  | 0.069*** |
| Nor born in country |  | -0.013** |  | -0.001 |
| **Education** |  |  |  |  |
| High education |  | (Baseline) |  | (Baseline) |
| Intermediate education |  | 0.044*** |  | 0.061*** |
| Low education |  | 0.101*** |  | 0.138*** |
| *Observations* | *182,195* | *182,195* | *2,412,791* | *2,412,791* |

^1^ Years differ in ESS and EU-SILC due to data availability: periods are split into early (2002-4 ESS, 2004 EU-SILC), recent (2012-2014), and an intermediate period (2005-2011 EU-SILC, 2006-2010 ESS).

## Appendix A3: Sensitivity analyses

The main text notes, *“These findings are robust to a variety of sensitivity analyses, including different definitions of employment, using a continuous rather than categorical trend term, using multilevel models rather than cluster-robust standard errors, and using logit rather than OLS.”* This appendix provides full results of these various sensitivity analyses in turn.

#### Different definitions of employment

In ESS, the main analysis defines someone as employed if they give the answer *‘in paid work (or away temporarily) (employee, self-employed, working for your family business)’*.[[3]](#footnote-4) This is similar to the EU-SILC definition of employment, but slightly different to the EU-LFS measure of 1+ hrs of work in the past seven days (see Appendix A1).

We therefore run a further sensitivity analysis on an alternative definition of work in ESS, based on the follow-up question, *“Can I just check, did you do any paid work of an hour or more in the last seven days?”.* As can be seen in Table A5 below, the results are effectively identical using either definition of employment.

Table A5: Trends in employment among disabled people 2002-2014 in ESS,

using an alternative definition of work

|  | **Main definition** | | **Alternate definition** | |
| --- | --- | --- | --- | --- |
|  | Model 1 | Model 2 | Model 3 | Model 4 |
| No disability | 0.280*** | 0.210*** | 0.281*** | 0.207*** |
| **Trends among disabled people^1^** |  |  |  |  |
| Pre-2006 | (Baseline) | (Baseline) | (Baseline) | (Baseline) |
| 2006-2011 | 0.051*** | 0.040** | 0.048*** | 0.038** |
| Post-2011 | 0.089*** | 0.076*** | 0.087*** | 0.075*** |
| **Trends in disability employment gap^1^** |  |  |  |  |
| No disability * 2006-2011 | -0.013 | -0.008 | -0.014 | -0.009 |
| No disability * Post-2011 | -0.057*** | -0.049*** | -0.057*** | -0.048*** |
| **Controls^2^** | No | Yes | No | Yes |
| *Observations* | *182,195* | *182,195* | *182,195* | *182,195* |

^1^ Years in ESS are pre-2004 (2002-4), recent (2012-2014), and an intermediate period (2006-2010 ESS). ^2^ Controls are age, gender, education, migrant status, living with partner, and any children in the household.

#### Continuous rather than categorical trends

The main models periodise the trends into pre-2006, 2006-2011, and post-2011. An alternate way of looking at trends is to look at a linear effect of year, and this is shown in Table A6 and A7 below. Using this alternate specification, we come to identical conclusions as in the main paper (that is, the disability employment gap declined in ESS but not EU-SILC, while disability rose in ESS but not EU-SILC).

Table A6: Trends in employment among disabled people 2002-2014,

using linear rather than categorical year for the trend

|  | **ESS** | | **EU-SILC** | |
| --- | --- | --- | --- | --- |
|  | Model 1 | Model 2 | Model 3 | Model 4 |
| No disability | 0.289*** | 0.218*** | 0.293*** | 0.218*** |
| **Trends among disabled people** |  |  |  |  |
| Year | 0.008*** | 0.007*** | 0.005** | 0.006*** |
| **Trends in disability employment gap^1^** |  |  |  |  |
| No disability * Year | -0.005*** | -0.004** | -0.001 | -0.003 |
| **Controls^1^** | No | Yes | No | Yes |
| *Observations* | *182,195* | *182,195* | *2,412,791* | *2,412,791* |

^1^ Controls are age, gender, education, migrant status, living with partner, and any children in the household.

Table A7: Trends in employment among disabled people 2002-2014,

using linear rather than categorical year for the trend

|  | **ESS** | | **EU-SILC** | |
| --- | --- | --- | --- | --- |
|  | Model 1 | Model 2 | Model 3 | Model 4 |
| Year | 0.002*** | 0.002*** | 0.001 | 0.001 |
| Controls^1^ | No | Yes | No | Yes |
| *Observations* | *182,195* | *182,195* | *2,412,791* | *2,412,791* |

^1^ Controls are age, gender, education, migrant status, living with partner, and any children in the household.

#### Logit models vs. OLS for binary outcomes

In the main text, we say, *“Because logit models are often misinterpreted [4], and linear regression for common binary outcomes is equally robust [5], we use linear (OLS) regression models; logit sensitivity analyses are supplied in Appendix A3 and show effectively identical results.”* These models are shown in Table A8 and A9 below (note that the coefficients are on different scales so that their absolute size should not be compared; we here focus on the relative size of different effects and their statistical significance).

There is a slight difference between logit and OLS models for EU-SILC, in that there is evidence that the absolute employment rate of disabled people increased when using logit but not OLS. However, the main finding – that ESS shows a declining disability employment gap, whereas EU-SILC shows no change in the disability employment gap – is unchanged.

Table A8: Trends in employment among disabled people 2002-2014,

comparing OLS vs. logit models for ESS

|  | **OLS** | | **Logit** | |
| --- | --- | --- | --- | --- |
|  | Model 1 | Model 2 | Model 3 | Model 4 |
| No disability | 0.280*** | 0.210*** | 1.187*** | 1.047*** |
| **Trends among disabled people** |  |  |  |  |
| Pre-2006 | (Baseline) | (Baseline) | (Baseline) | (Baseline) |
| 2006-2011 | 0.051*** | 0.040** | 0.206*** | 0.198*** |
| Post-2011 | 0.089*** | 0.076*** | 0.362*** | 0.369*** |
| **Trends in disability employment gap^1^** |  |  |  |  |
| No disability * 2006-2011 | -0.013 | -0.008 | -0.014 | -0.006 |
| No disability * Post-2011 | -0.057*** | -0.049*** | -0.203** | -0.212** |
| **Controls^1^** | No | Yes | No | Yes |
| *Observations* | *182,195* | *182,195* | *2,412,791* | *2,412,791* |

Note: table displays logit coefficients and not odds ratios. ^1^ Controls are age, gender, education, migrant status, living with partner, and any children in the household.

Table A9: Trends in employment among disabled people 2002-2014,

comparing OLS vs. logit models for EU-SILC

|  | **OLS** | | **Logit** | |
| --- | --- | --- | --- | --- |
|  | Model 1 | Model 2 | Model 3 | Model 4 |
| No disability | 0.263*** | 0.183*** | 1.209*** | 1.021*** |
| **Trends among disabled people^1^** |  |  |  |  |
| Pre-2006 | (Baseline) | (Baseline) | (Baseline) | (Baseline) |
| 2006-2011 | -0.008 | -0.013 | 0.033 | 0.065 |
| Post-2011 | 0.027 | 0.026 | 0.136* | 0.195** |
| **Trends in disability employment gap^1^** |  |  |  |  |
| No disability * 2006-2011 | 0.030* | 0.030* | 0.120 | 0.093 |
| No disability * Post-2011 | 0.011 | 0.004 | 0.009 | -0.066 |
| **Controls^1^** | No | Yes | No | Yes |
| *Observations* | *182,195* | *182,195* | *2,412,791* | *2,412,791* |

Note: table displays logit coefficients and not odds ratios. ^1^ Controls are age, gender, education, migrant status, living with partner, and any children in the household.

#### Multilevel models vs. cluster-robust single-level models

In the main text, we say *“because the partitioning of variance between the individual and societal level is not of interest here, we use cluster-robust regression models; multilevel models in Appendix A3 again give identical results.”* These models are shown in Tables A10 and A11 below.

Again, there is a slight difference between the cluster-robust single-level and multilevel models for EU-SILC, in that the trends in the disability employment gap in EU-SILC are significant in the multilevel models but not the single-level models. (This is particularly surprising given that in simulations, it is multilevel models that have been shown to be more conservative [6]). However, there are two ways in which differences between EU-SILC & ESS remain:

- The narrowing disability employment gap in EU-SILC is only visible after controlling for compositional changes – in the raw data, we instead see an *increase* in the disability employment gap (Table A11 model 3).
- The size of the changing disability employment gap in EU-SILC is relatively small (by less than 1 percentage point), about one-third of the size of the 2 percentage point narrowing in ESS.

Table A10: Trends in employment among disabled people 2002-2014,

comparing unweighted cluster-robust single-level vs. unweighted multilevel models for ESS

|  | **OLS** | | **Multilevel** | |
| --- | --- | --- | --- | --- |
|  | Model 1 | Model 2 | Model 3 | Model 4 |
| No disability | 0.275*** | 0.209*** | 0.272*** | 0.206*** |
| **Trends among disabled people** |  |  |  |  |
| Pre-2006 | (Baseline) | (Baseline) | (Baseline) | (Baseline) |
| 2006-2011 | 0.022* | 0.021* | 0.018** | 0.016** |
| Post-2011 | 0.049*** | 0.045*** | 0.042*** | 0.037*** |
| **Trends in disability employment gap^1^** |  |  |  |  |
| No disability * 2006-2011 | 0.001 | -0.003 | 0.004 | 0.001 |
| No disability * Post-2011 | -0.025* | -0.027** | -0.019*** | -0.020*** |
| **Controls^1^** | No | Yes | No | Yes |
| *Observations* | *182,195* | *182,195* | *2,412,791* | *2,412,791* |

^1^ Controls are age, gender, education, migrant status, living with partner, and any children in the household.

Table A11: Trends in employment among disabled people 2002-2014,

comparing unweighted cluster-robust single-level vs. unweighted multilevel models for EU-SILC

|  | **OLS** | | **Multilevel** | |
| --- | --- | --- | --- | --- |
|  | Model 1 | Model 2 | Model 3 | Model 4 |
| No disability | 0.274*** | 0.190*** | 0.280*** | 0.194*** |
| **Trends among disabled people^1^** |  |  |  |  |
| Pre-2006 | (Baseline) | (Baseline) | (Baseline) | (Baseline) |
| 2006-2011 | -0.002 | 0.006 | -0.008 | 0.001 |
| Post-2011 | 0.005 | 0.017 | -0.007 | 0.004 |
| **Trends in disability employment gap^1^** |  |  |  |  |
| No disability * 2006-2011 | 0.024 | 0.015 | 0.018*** | 0.010*** |
| No disability * Post-2011 | 0.008 | -0.007 | 0.004* | -0.007*** |
| **Controls^1^** | No | Yes | No | Yes |
| *Observations* | *182,195* | *182,195* | *2,412,791* | *2,412,791* |

^1^ Controls are age, gender, education, migrant status, living with partner, and any children in the household.

## Bibliography for Appendix

1. Croezen S, Burdorf A, van Lenthe FJ: Self-perceived health in older Europeans: Does the choice of survey matter? *The European Journal of Public Health* 2016, 26:686-692.

2. Berthoud R: Trends in the Employment of Disabled People in Britain. In *ISER Working Paper No 2011-03*: Institute of Social and Economic Research (ISER), University of Essex; 2011.

3. Jones M, Wass V: Understanding changing disability-related employment gaps in Britain 1998–2011. *Work, Employment & Society* 2013, 27:982-1003.

4. Mood C: Logistic Regression: Why We Cannot Do What We Think We Can Do, and What We Can Do About It. *European Sociological Review* 2010, 26:67-82.

5. Hellevik O: Linear versus logistic regression when the dependent variable is a dichotomy. *Quality & Quantity* 2009, 43:59-74.

6. Cheah BC: Clustering Standard Errors or Modeling Multilevel Data? In *Unpublished working paper*. New York: Columbia University; 2009.

1. <http://www.unesco.org/education/information/nfsunesco/doc/isced_1997.htm> [↑](#footnote-ref-2)
2. <http://www.europeansocialsurvey.org/docs/methodology/education_upgrade_ESS1-4_e01_3.pdf> [↑](#footnote-ref-3)
3. This is a response to the question, *“Using this card, which of these descriptions applies to what you have been doing for the last 7 days? Select all that apply.”* [↑](#footnote-ref-4)
